# An electrochemical study of pH influences on corrosion and passivation for a Q235 carbon steel in HNO_3_–NaNO_2_, HAc–NaNO_2_ and HCl–NaNO_2_ solutions

**DOI:** 10.1039/c9ra08482g

**Published:** 2019-11-28

**Authors:** Xuan Li, Pei Zhang, Huiju Huang, Xiaochen Hu, Yong Zhou, Fuan Yan

**Affiliations:** Key Laboratory for Green Chemical Process of Ministry of Education, Wuhan Institute of Technology Wuhan 430205 China zhouyong@wit.edu.cn; College of Chemistry and Food Science, Yulin Normal University Yulin 537000 China

## Abstract

In this study, the influences of different pH values on the corrosion and passivation behaviors of a Q235 carbon steel in HNO_3_–NaNO_2_, HAc–NaNO_2_ and HCl–NaNO_2_ solutions were studied by electrochemical methods. The manifestations of the electrochemical characteristics were revealed and the variations in the electrochemical parameters were clarified. Moreover, for the Q235 steel in the three solutions with different pH values, the decrease in the corrosion current density (*i*_corr_) and the increase in the charge transfer resistance (*R*_ct_) in each solution, indicated a decrease in the corrosion rate. The decrease in the critical passivation current density (*i*_crit_) and increase in the passive film resistance (*R*_f_) suggested the reinforcement of passivation capability. On the other hand, in the three solutions at the same pH value, the corrosion rate increased and the passivation capability weakened in HNO_3_–NaNO_2_, HAc–NaNO_2_ and HCl–NaNO_2_ solutions. Simultaneously, the related electrochemical mechanisms of corrosion and passivation for Q235 carbon steel in acidic solutions containing nitrite anions (NO_2_^−^) were also discussed.

## Abstract 2

In this study, the influences of different pH values on the corrosion and passivation behaviors of a Q235 carbon steel in HNO_3_–NaNO_2_, HAc–NaNO_2_ and HCl–NaNO_2_ solutions were studied by electrochemical methods.

## Graphical abstract

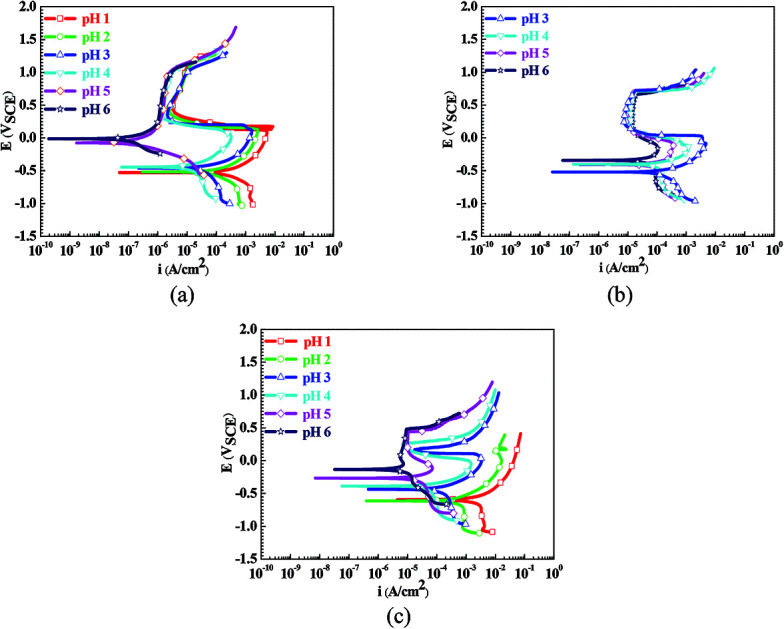

## 1. Introduction

Carbon steel materials are widely used in the construction of large engineering structures.^1–3^ However, in electrolytic environments, the corrosion of structural steel products is unavoidable,^4^ especially in strong electrolytes.^5^ The addition of corrosion inhibitors, mainly including oxidation and/or adsorption species, into electrolytic environments can inhibit the corrosion process of metals and alloys effectively.^6^

As an oxidizing type inhibitor, the addition of nitrite anions (NO_2_^−^) into alkaline and neutral electrolytes significantly decreases the corrosion rate for carbon steels, which is attributed to the function of NO_2_^−^ on the repassivation of the steel surface,^7–12^ and the crucial electrochemical mechanism of NO_2_^−^ is as follows:

1 2Fe^2+^ + 2OH^−^ + 2NO_2_^−^ → 2NO + γ-Fe_2_O_3_ + H_2_O

At present, in alkaline and neutral electrolytes containing NO_2_^−^, many studies concerning the corrosion and passivation behaviors of carbon steels have been reported.^7–12^

In contrast, there are relatively few related studies in acidic electrolytes containing NO_2_^−^.^13–17^ Zhou *et al.*^13^ studied the corrosion and passivation behaviors of Q235 carbon steel in CO_2_ saturated solutions containing NO_2_^−^. The authors reported that the electrochemical characteristics of the Q235 steel transferred from the active dissolution in the single CO_2_ solution free of NO_2_^−^ to the anodic passivation in the corresponding solutions containing NO_2_^−^, which was due to the formation of the Fe_2_O_3_ passive film under the FeCO_3_ layer. Zuo *et al.*^14^ studied the pitting and passivation behaviors of the X70 carbon steel in acidic NaCl solutions containing NO_2_^−^ and thioureido imidazoline (TAI). The authors reported that although the interactive and superimposed mechanism between NO_2_^−^ and TAI was present, NO_2_^−^ (rather than TAI) was responsible for the passivation of the X70 steel. However, in the studies of Zhou *et al.*^13^ and Zuo *et al.*,^14^ the pH value was kept at pH 3.7 and pH 5.5, respectively, which is too limited in scope and not systematic. In contrast, Garces *et al.*^16^ studied the corrosion and passivation behaviors of a corrugated steel bar in simulated pit solutions containing NO_2_^−^ from pH 1.46 to pH 6.38. For the corrugated steel bar in the simulated solutions, the authors reported that the addition of NO_2_^−^ promoted the surface passivation and restrained the pitting corrosion, but the general corrosion rate also increased. Zhou *et al.*^17^ studied the corrosion and passivation behaviors of Q235 carbon steel in HCl solutions containing NO_2_^−^ from pH 1 to pH 6. The authors reported that uniform corrosion, intergranular corrosion and pitting corrosion occurred on the surface of the Q235 carbon steel in sequence with the increasing pH value, and NO_2_^−^ played a critical role in the above three corrosion stages. Nevertheless, in the studies of Garces *et al.*^16^ and Zhou *et al.*,^17^ the influences of the pH value and NO_2_^−^ presence on the variations in the electrochemical parameters were not discussed in detail.

In a previous study,^18^ we carried out potentiodynamic polarization tests on the L80, N80, X65 and Q235 steels in HNO_3_–NaNO_2_, HCl–NaNO_2_, HAc–NaNO_2_ and CO_2_–NaNO_2_ solutions and reported the relationship between the activation–passivation transition and the grain boundary dissolution for both carbon steels and alloy steels in acidic solutions containing NO_2_^−^. However, in the above study, the detailed pH influences on the corrosion and passivation behaviors, particularly on the manifestations of electrochemical characteristics and the variations of electrochemical parameters, were not discussed. Therefore, in this study, the influence of different pH values on the corrosion and passivation behaviors of Q235 carbon steel in HNO_3_–NaNO_2_, HAc–NaNO_2_ and HCl–NaNO_2_ solutions are studied by electrochemical methods, and the related electrochemical mechanisms are also discussed in detail.

## 2. Experimental

The studied material was Q235 carbon steel with the following chemical composition (weight percent): C, 0.160; Mn, 0.530; Si, 0.300; S, 0.045; P, 0.015, and Fe, balance. Samples were manually abraded up to 1000 grit with SiC abrasive papers, rinsed with de-ionized water and degreased in alcohol.

The studied solutions were HNO_3_–NaNO_2_, HAc–NaNO_2_ and HCl–NaNO_2_ solutions. The diluted HNO_3_, HAc and HCl solutions were each introduced into a 0.01 mol L^−1^ NaNO_2_ solution to adjust the pH value and obtain the three final solutions.

The electrochemical tests of potentiodynamic polarization, cyclic voltammetry, electrochemical impedance spectroscopy (EIS) and potential step were performed at ambient temperature by a CS310 electrochemical workstation (China). A typical three-electrode system was used for all electrochemical tests. The system was composed of a saturated calomel electrode (SCE) or a silver–silver chloride electrode (Ag/AgCl) as the reference electrode, a platinum sheet as the counter electrode and a Q235 sample as the working electrode. The SCE electrode was applied in the electrochemical tests of potentiodynamic polarization, EIS and potential step; the Ag/AgCl electrode was applied in the cyclic voltammetry tests. Before each electrochemical test, the working electrode was immersed in the corresponding studied solution for a certain period of time until the open circuit potential (OCP) was stable. In the potentiodynamic polarization tests, the potential scanning rate was 0.5 mV s^−1^, and the potential scanning range was from −0.5 V_OCP_ to the potential value corresponding to the objective electrochemical characteristic. In the cyclic voltammetry tests, the potential scanning rate was 30 mV s^−1^, and the potential scanning range was from −2.0 V_Ag/AgCl_ to 2.0 V_Ag/AgCl_. In the EIS tests, a perturbation potential of 10 mV amplitude was used in the frequency range from 10^5^ to 10^−2^ Hz. In the potential step tests, an applied potential was suddenly added on the working electrode, and the recording frequency of the current density was 5 Hz.

## 3. Results and discussion

### 3.1 Electrochemical characteristic

**Fig. 1:**
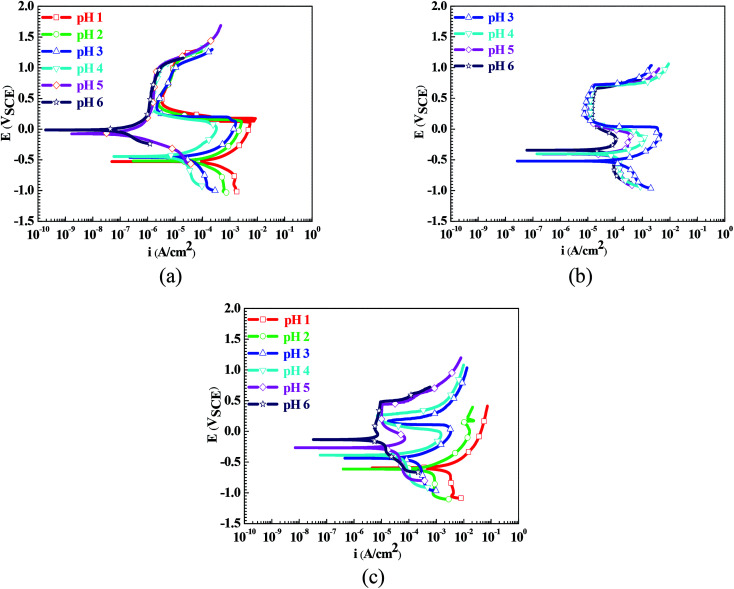
Polarization curves of the Q235 samples in HNO_3_–NaNO_2_, HAc–NaNO_2_ and HCl–NaNO_2_ solutions: (a) HNO_3_–NaNO_2_ solutions, (b) HAc–NaNO_2_ solutions and (c) HCl–NaNO_2_ solutions.

Fig. 1 shows the polarization curves of the Q235 samples in the HNO_3_–NaNO_2_, HAc–NaNO_2_ and HCl–NaNO_2_ solutions. From Fig. 1, the influences of the pH values on the electrochemical characteristics of the Q235 carbon steel in the three solutions are very prominent. In the HNO_3_–NaNO_2_ solutions of pH 1–4 and in the HAc–NaNO_2_ solutions of pH 3–6, the Q235 samples show a similar electrochemical characteristic: the anodic current density first increased gradually with the positive shift of applied potential, but then decreased suddenly when the applied potential reached the activation–passivation transition potential (*E*_trans_) value corresponding to the critical passivation current density (*i*_crit_); after that, the anodic current density remained at the maintaining passivation current density (*i*_main_) until the occurrence of transpassivation. The above electrochemical characteristic is called “activation–passivation–transpassivation (A–P–T)” in this work. The anodic current density also first increased gradually with the positive shift of applied potential in the HNO_3_–NaNO_2_ solutions at pH 5 and pH 6, but directly reached the *i*_main_ value without the activation–passivation transition, which is called “self-passivation–transpassivation (sp-T)”. In the HCl–NaNO_2_ solutions at pH 1 and pH 2, the anodic current density increased continuously with the positive shift of applied potential. This is called “activation (A)” in this work. In the HCl–NaNO_2_ solutions at pH 3 to pH 6, the initial evolution of the anodic current density in these solutions was very similar to that in the HNO_3_–NaNO_2_ solutions at pH 1 to pH 4. However, due to the occurrence of pitting corrosion induced by Cl^−^,^19^ the anodic current density increased suddenly when the applied potential reached the pitting potential (*E*_pit_). This electrochemical characteristic is called “activation-passivation-pitting (A–P–P)”.

When the scanning of the applied potential is close to the corrosion potential (*E*_corr_) for carbon steels in acidic electrolytes, the main anodic reaction is the Fe oxidation with a standard potential (*E*_st_) of −0.441 V_SHE_,^20^ which is given as follows:

2 Fe → Fe^2+^ + 2e (*E*_st_ = −0.441 V_SHE_)

At the same time, the main cathodic reactions depend on the pH value of the acidic electrolytes. The H^+^ reduction and the O_2_ reduction occur at relatively low and high pH values,^15^ respectively, which are given as follows:

3 2H^+^ + 2e → H_2_ (*E*_st_ = 0 V_SHE_)

4 O_2_ + 2H_2_O + 4e → 4OH^−^ (*E*_st_ = 0.401 V_SHE_)

It is generally accepted that carbon steels usually show an electrochemical characteristic of active dissolution in acidic electrolytes.^21^ The manifestation on the polarization curve is that the anodic current density keeps increasing with the positive shift of the applied potential.^22^ In this work, similar results were also observed on the polarization curves when the scanning of the applied potential slightly exceeded *E*_corr_, as shown in Fig. 1.

However, except in the pH 1 and pH 2 HCl–NaNO_2_ solutions, a passivation occurrence was present on the polarization curves with a positive shift of the applied potential, as shown in Fig. 1. One is the anodic passivation like the polarization curves in the HNO_3_–NaNO_2_ solutions at pH 1 to pH 4, in the HAc–NaNO_2_ solutions at pH 3 to pH 6 and in the HCl–NaNO_2_ solutions at pH 3 to pH 6: the anodic current density decreased suddenly when the applied potential reached the *E*_trans_ value. The other is the spontaneous passivation like the polarization curves in the HNO_3_–NaNO_2_ solutions at pH 5 and pH 6: the anodic current density remained at the *i*_main_ value before the applied potential reached the transpassivation potential. A large number of studies have reported that the oxidation from Fe^2+^ to Fe^3+^ plays a critical role in the surface passivation of steel materials.^23–25^ The anodic reaction of Fe^2+^ oxidation is given as follows:

5 Fe^2+^ → Fe^3+^ + e (*E*_st_ = 0.771 V_SHE_)

**Fig. 2:**
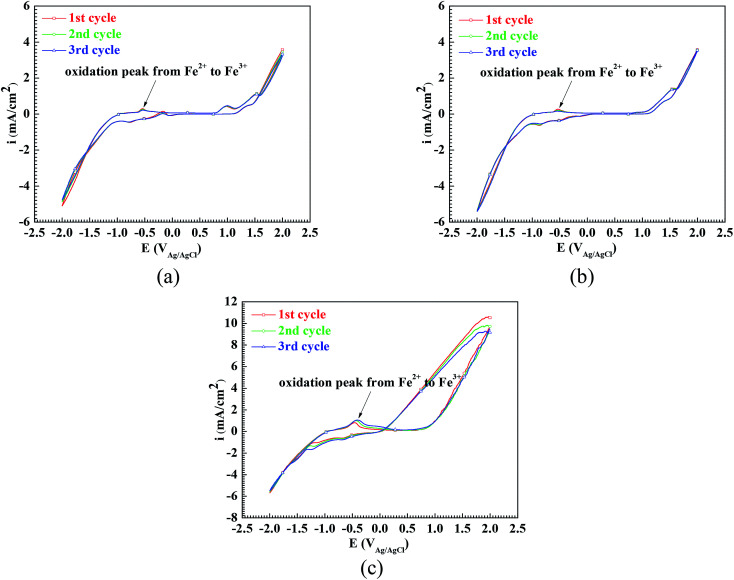
Cyclic voltammograms of the Q235 samples in HNO_3_–NaNO_2_, HAc–NaNO_2_ and HCl–NaNO_2_ solutions at pH 4: (a) HNO_3_–NaNO_2_ solution, (b) HAc–NaNO_2_ solution and (c) HCl–NaNO_2_ solution.

In order to confirm the oxidation from Fe^2+^ to Fe^3+^ on the surface of the Q235 carbon steel in the three solutions, the electrochemical tests of cyclic voltammetry were carried out. Fig. 2 shows the cyclic voltammograms of the Q235 samples in the HNO_3_–NaNO_2_, HAc–NaNO_2_ and HCl–NaNO_2_ solutions at pH 4. The reason why the pH 4 solutions are chosen is that the cathodic reactions involving NO_2_^−^ reduction and their equilibrium potential at pH 4 have been reported by Li *et al.*^26^ From Fig. 2, for the Q235 steel in the pH 4 solutions, the CV peaks corresponding to the oxidation from Fe^2+^ to Fe^3+^^27–29^ are observed on the cyclic voltammograms. In pure acidic electrolytes free of oxidants, the anodic reaction of Fe^2+^ oxidation is not available, which is attributed to the *E*_st_ value of Fe^2+^ oxidation (0.771 V_SHE_) being greater than that of H^+^ reduction (0 V_SHE_) or O_2_ reduction (0.401 V_SHE_). However, in this work, due to the presence of NO_2_^−^ in the three solutions, the cathodic reaction of NO_2_^−^ reduction made the anodic reaction of Fe^2+^ oxidation possible.^13,15,18^ It is noteworthy from Fig. 2 that the CV peaks corresponding to the reduction of NO_2_^−^ were absent, and similar results were also obtained by Li *et al.*^26^ and Valcarce *et al.*^11^ Regarding the cathodic reduction of NO_2_^−^, Zhou *et al.*^13^ reported the following electrode reaction in a CO_2_-saturated solution:

6 NO_2_^−^ + e → NO + O^2−^ (*E*_st_ = 0.715 V_SCE_ or 0.959 V_SHE_)

In addition, Li *et al.*^26^ reported the following electrode reactions:

7 2NO_2_^−^ + 8H^+^ + 6e → N_2_ + 4H_2_O (*E*_st_ = 0.944 V_SCE_ or 1.188 V_SHE_)

8 2NO_2_^−^ + 6H^+^ + 4e → N_2_O + 3H_2_O (*E*_st_ = 0.771 V_SCE_ or 1.015 V_SHE_)

Because there was a cathodic reaction whose *E*_st_ was more positive than the *E*_st_ of Fe^2+^ oxidation, the anodic reaction of Fe^2+^ oxidation was possible, resulting in the occurrence of passivation on the surface of the Q235 carbon steel.

From the above discussion, for the Q235 carbon steel in the three solutions, the corrosion occurred when the applied potential was close to *E*_corr_, followed by the occurrence of passivation with the gradual positive shift of the applied potential. The influences of pH values on the electrochemical parameters of corrosion and passivation are significant, which will be discussed as follows. In this work, in order to obtain the electrochemical parameters, the polarization curves were fitted by the CVIEW software according to the Tafel interpretation, and the EIS were fitted by the ZVIEW software according to the equivalent electrical circuit (EEC) interpretation.

### 3.2 Corrosion behavior (corrosion rate)

**Fig. 3:**
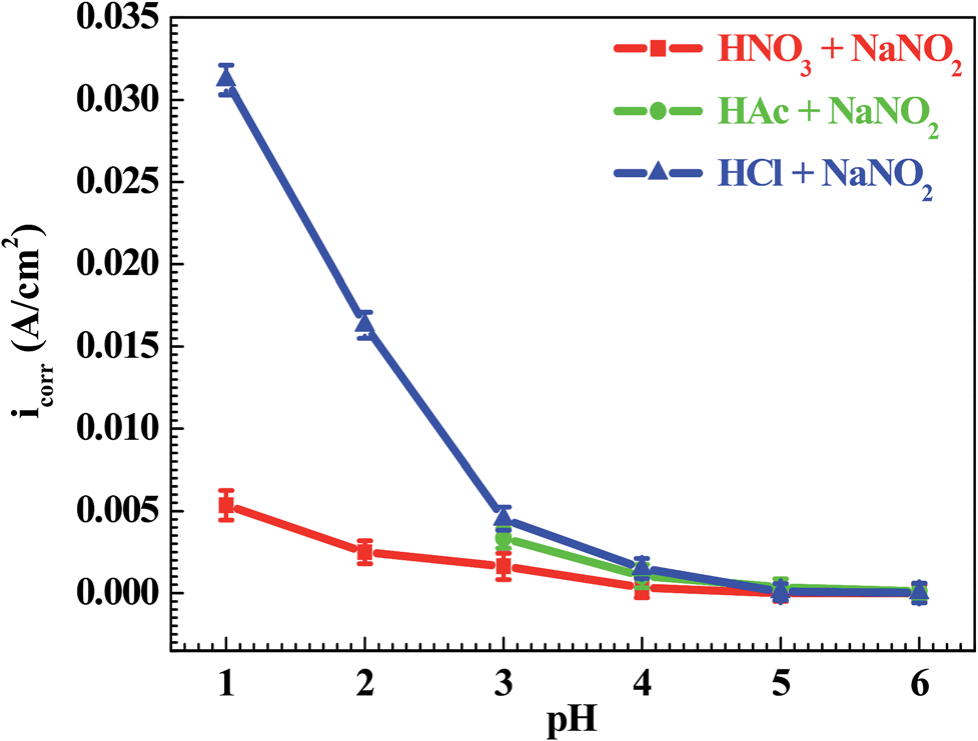
pH influences on the values of the corrosion current density (*i*_corr_).

Fig. 3 shows the pH influences on the values of the corrosion current density (*i*_corr_). From Fig. 3, the *i*_corr_ value in each solution decreased with increasing pH value, indicating the pH influence on the corrosion rate. The chemical equilibriums of H^+^ reduction (eqn (3)) and O_2_ reduction (eqn (4)) moved toward the left direction with increasing pH value, resulting in a decrease of the corrosion rate.^30^ At the same time, the *i*_corr_ value gradually increased in the HNO_3_–NaNO_2_, HAc–NaNO_2_ and HCl–NaNO_2_ solutions at the same pH value (Fig. 3), which is attributed to the inhibitive and aggressive properties of NO_3_^−^,^31^ and Cl^−^,^32^ respectively.

**Fig. 4:**
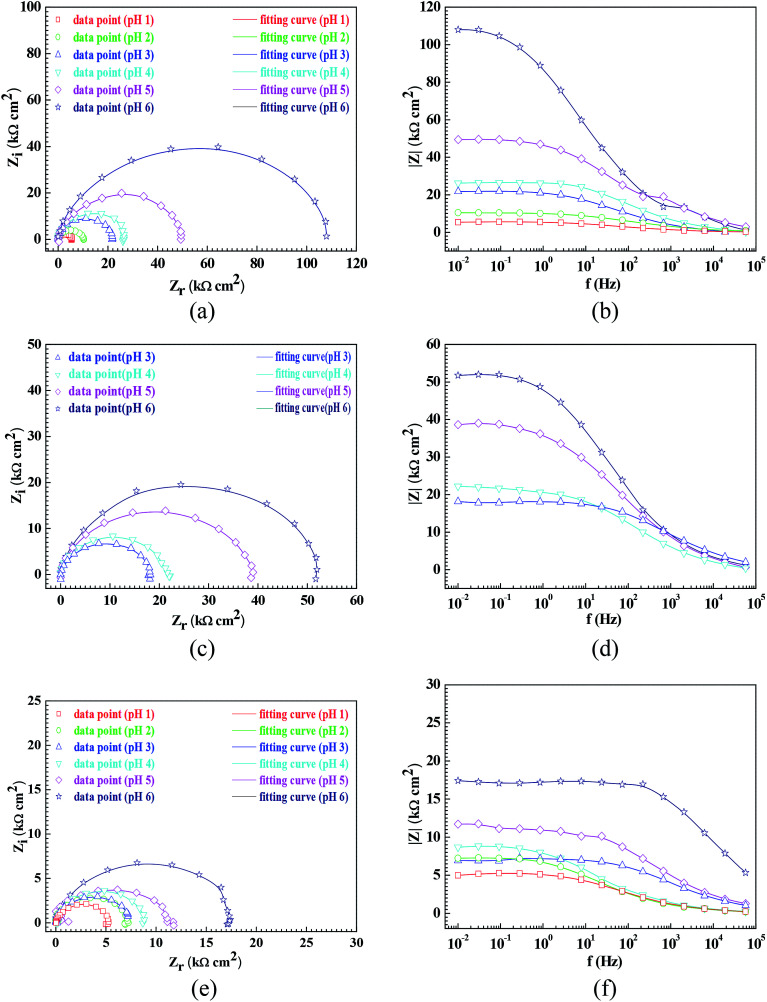
Nyquist and Bode plots of the Q235 samples in the HNO_3_–NaNO_2_, HAc–NaNO_2_ and HCl–NaNO_2_ solutions at an applied potential of OCP: (a) Nyquist plots in HNO_3_–NaNO_2_ solutions, (b) Bode plots in HNO_3_–NaNO_2_ solutions, (c) Nyquist plots in HAc-NaNO_2_ solutions, (d) Bode plots in HAc–NaNO_2_ solutions, (e) Nyquist plots in HCl–NaNO_2_ solutions and (f) Bode plots in HCl–NaNO_2_ solutions.

**Fig. 5:**
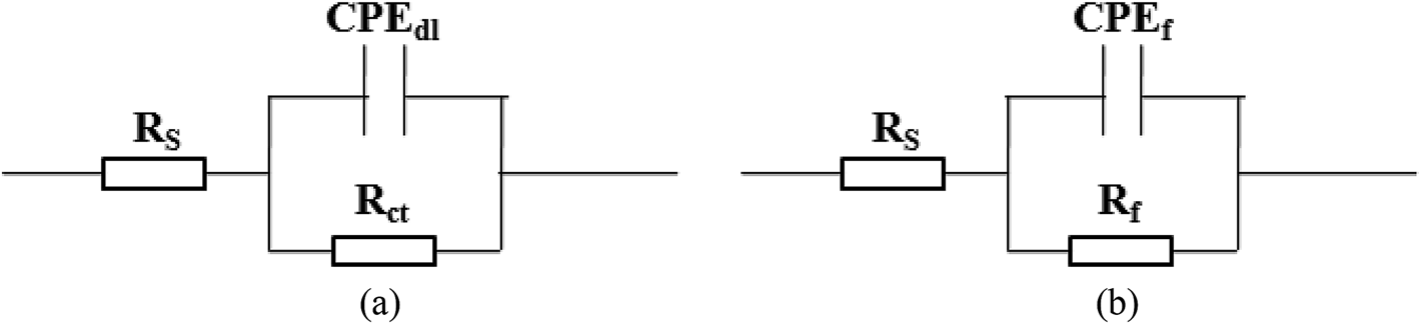
Equivalent electrical circuit (EEC) models for EIS interpretation: (a) EIS shown in Fig. 4 and (b) EIS shown in Fig. 8.

**Fig. 6:**
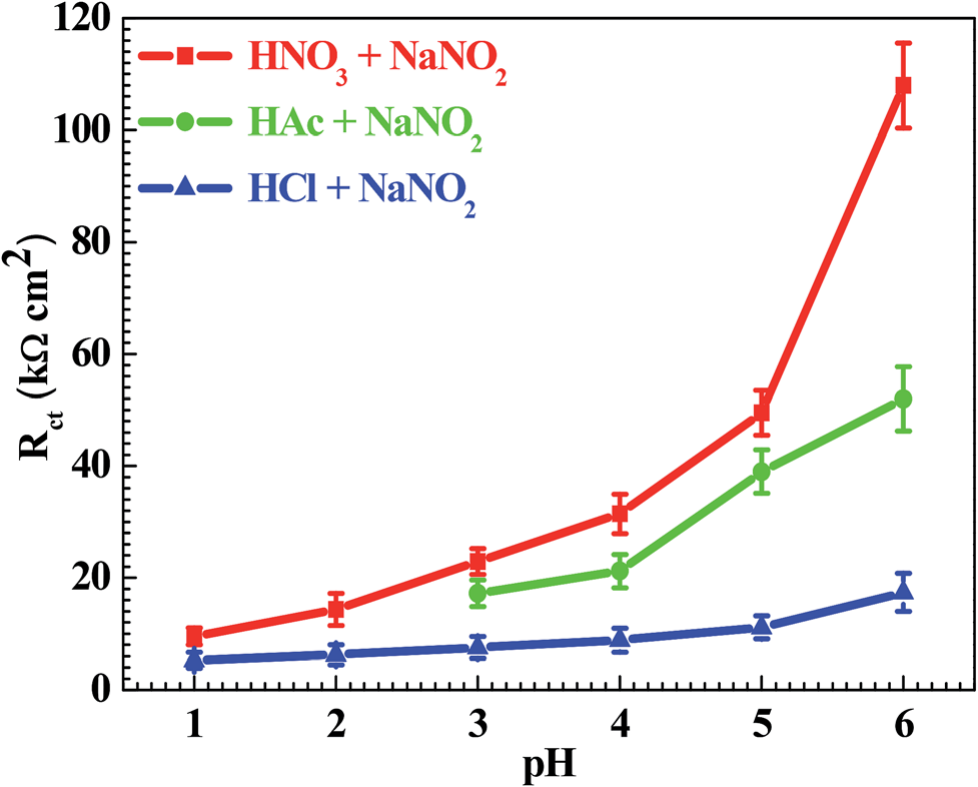
pH influences on the values of the charge transfer resistance (*R*_ct_).

In order to further confirm the influence of the pH value on the corrosion rate, EIS tests were also carried out. Fig. 4 shows the Nyquist and Bode plots of the Q235 samples in HNO_3_–NaNO_2_, HAc–NaNO_2_ and HCl–NaNO_2_ solutions at the applied potential of OCP. From the Nyquist plots shown in Fig. 4a, c and e, all Nyquist plots for the Q235 carbon steel in the three solutions at OCP are composed of a single depressed capacitive semicircle in the whole applied frequency range, which is independent of the pH value. It was reported that for carbon steels in acidic electrolytes, the single capacitive semicircle reflected the electrochemical process of a charge transfer between electron double layer.^33^ At the same time, the radius of the capacitive semicircle expanded with increasing pH value in each solution, indicating a decrease of the corrosion rate.^34^ On the other hand, from the Bode plots shown in Fig. 4b, d and f, the modulus at the low frequency limit of 0.01 Hz increased with increasing pH value in each solution, confirming the decrease of the corrosion rate. This result is in agreement with the *i*_corr_ result. Furthermore, the EIS results were interpreted with the EEC model shown in Fig. 5a, where *R*_S_ represents the solution resistance, CPE_dl_ represents the double layer capacitance, and *R*_ct_ represents the charge transfer resistance. It was reported that the *R*_ct_ value indicated the corrosion rate of the metals and alloys in electrolytic environments.^35^Fig. 6 shows the pH influences on the values of *R*_ct_. Comparing Fig. 6 with Fig. 3, the variations of *R*_ct_ and *i*_corr_ are very similar: in each solution, the *R*_ct_ value increased with increasing pH value. At the same pH value, the *R*_ct_ value in HNO_3_–NaNO_2_, HAc–NaNO_2_ and HCl–NaNO_2_ solutions gradually decreased in turn.

### 3.3 Passivation behavior (passivation capability)

From Fig. 1, the pH range for the surface passivation, including anodic passivation and spontaneous passivation, of the Q235 carbon steel in HNO_3_–NaNO_2_, HAc–NaNO_2_ and HCl–NaNO_2_ solutions was from pH 1 to pH 6, from pH 3 and pH 6 and from pH 3 to pH 6, respectively. On the one hand, the pH range of passivation occurrence in the HNO_3_–NaNO_2_ solutions was more extensive than that in the HAc–NaNO_2_ and HCl–NaNO_2_ solutions. On the other hand, the spontaneous passivation occurred in the HNO_3_–NaNO_2_ solutions at pH 5 and pH 6 only. The above two aspects indicate that for the Q235 carbon steel in the three solutions, the passivation capability in HNO_3_–NaNO_2_ solutions was strongest. Furthermore, the passivation capability of the Q235 steel in the HAc–NaNO_2_ solutions took second place, and the relatively narrow pH range of passivation occurrence was due to the weak acidic property of HAc.^18^ Although the surface passivation occurred in the HCl–NaNO_2_ solutions at pH 3 to pH 6, the formed passive film would inevitably be attacked by the aggressive Cl^−^ ions,^36^ so the passivation capability of the Q235 carbon steel in HCl–NaNO_2_ solutions would be the weakest. The above discussion will be confirmed by the *i*_crit_ results, passive film resistance (*R*_f_) and passivation power (*Q*_pass_) later. The influences of pH values on the main passivation parameters are discussed as follows, which only involve the electrochemical characteristics of A–P–T and A–P–P.

**Fig. 7:**
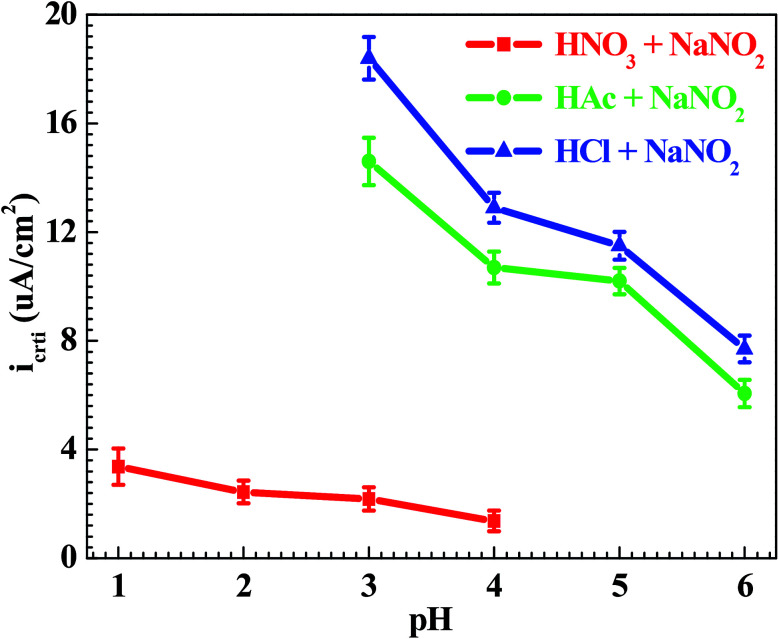
pH influences on the values of the critical passivation current density (*i*_crit_).

**Fig. 8:**
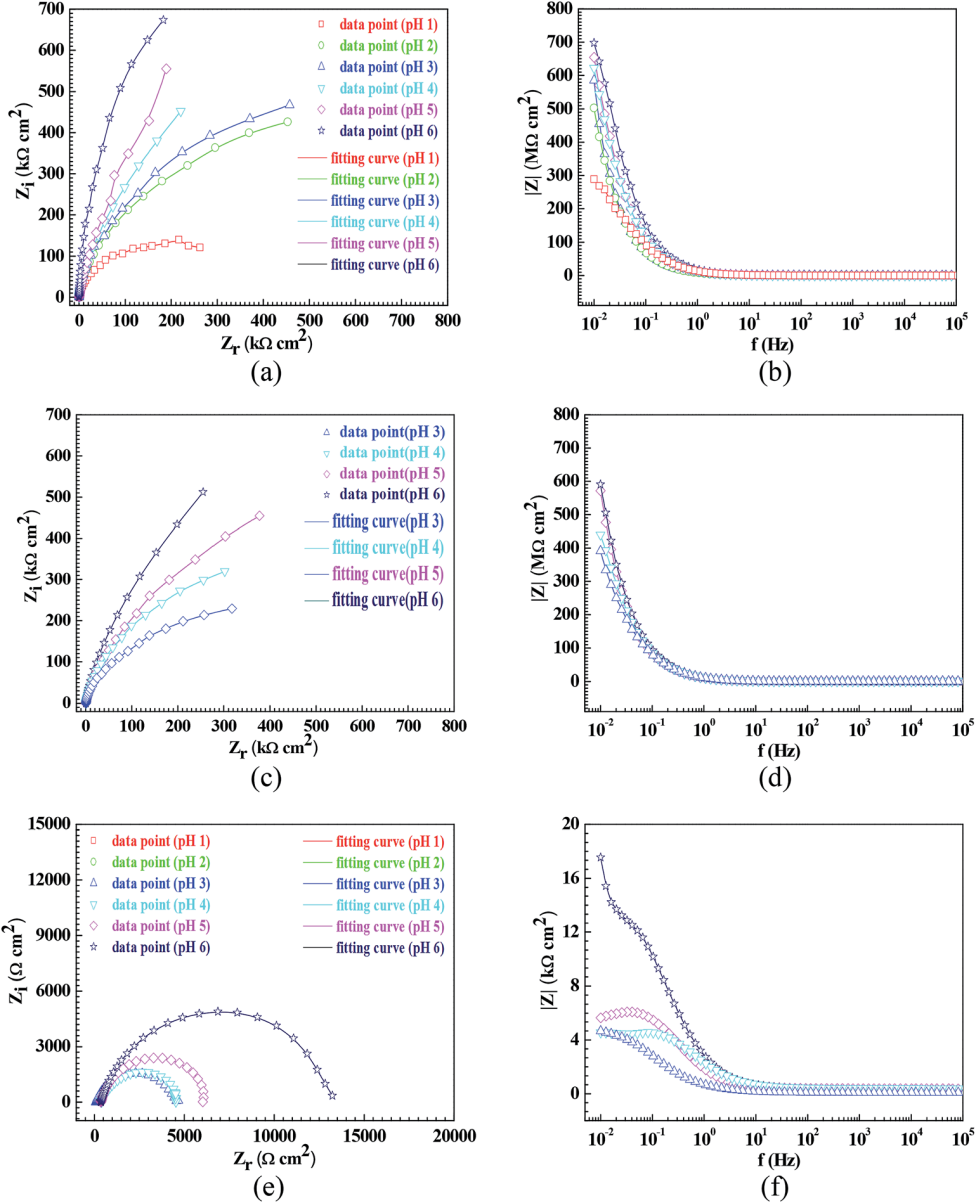
Nyquist and Bode plots of the Q235 samples in the HNO_3_–NaNO_2_, HAc–NaNO_2_ and HCl–NaNO_2_ solutions at an applied potential of *E*_inti_: (a) Nyquist plots in HNO_3_–NaNO_2_ solutions, (b) Bode plots in HNO_3_–NaNO_2_ solutions, (c) Nyquist plots in HAc–NaNO_2_ solutions, (d) Bode plots in HAc–NaNO_2_ solutions, (e) Nyquist plots in HCl–NaNO_2_ solutions and (f) Bode plots in HCl–NaNO_2_ solutions.

**Fig. 9:**
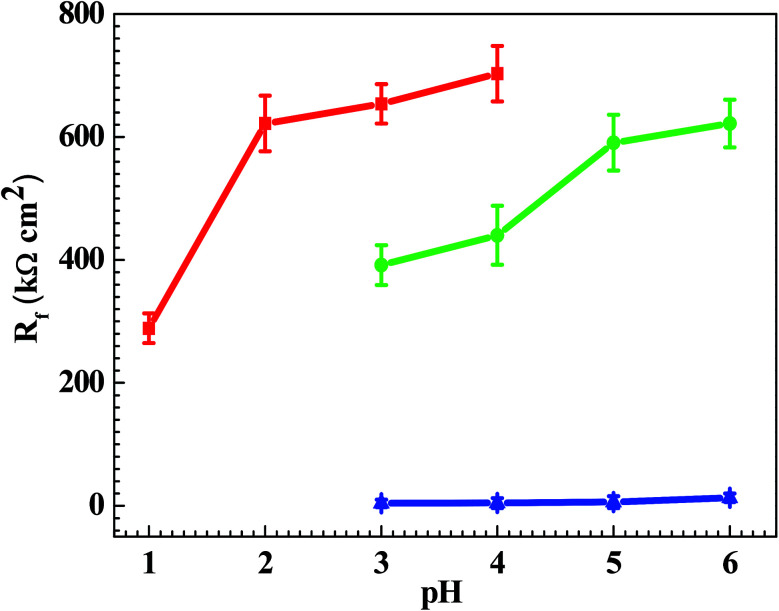
pH influences on the values of the passive film resistance (*R*_f_).

**Fig. 10:**
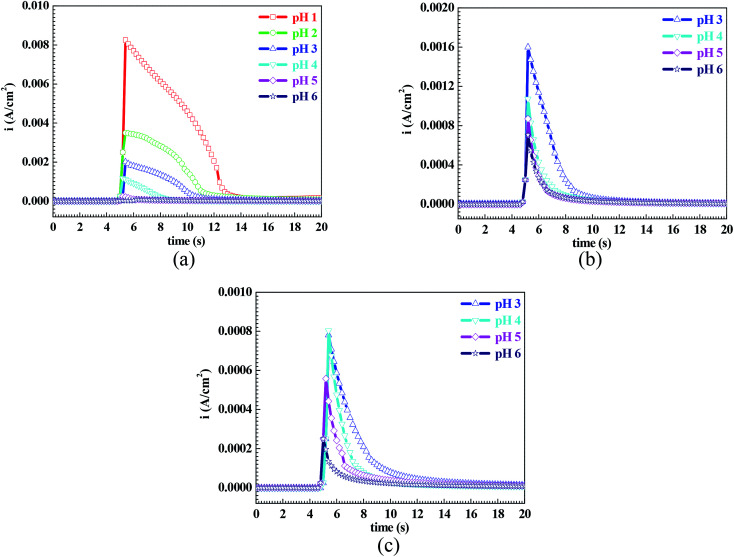
Current transients of the Q235 samples in the HNO_3_–NaNO_2_, HAc–NaNO_2_ and HCl–NaNO_2_ solutions at an applied potential of *E*_init_: (a) HNO_3_–NaNO_2_ solutions, (b) HAc–NaNO_2_ solutions and (c) HCl–NaNO_2_ solutions.

Fig. 7 shows the pH influences on the values of *i*_crit_. From Fig. 7, the *i*_crit_ value decreases in each solution with increasing pH value, suggesting the pH influence on the passivation capability. In order to further confirm the pH influence on the passivation capability, the initial passivation potential (*E*_init_) was added on the Q235 sample as an applied potential, and the electrochemical tests of EIS and potential step were carried out. Fig. 8 shows the Nyquist and Bode plots of the Q235 samples in the HNO_3_–NaNO_2_, HAc–NaNO_2_ and HCl–NaNO_2_ solutions at the applied *E*_init_ potential. From the Nyquist plots shown in Fig. 8a, c and e, the Nyquist plots of the Q235 carbon steel in the HNO_3_–NaNO_2_ and HAc–NaNO_2_ solutions were composed of an elevated capacitive semicircle, but those in the HCl–NaNO_2_ solutions were composed of a depressed capacitive semicircle, which may be attributed to the presence or absence of an aggressive species.^37^ At the same time, the radius of the capacitive semicircle in each solution expanded with increasing pH value, indicating the reinforcement of the passivation capability.^38^ On the other hand, from the Bode plots shown in Fig. 8b, d and f, the modulus at the low frequency limit of 0.01 Hz in each solution increased with increasing pH value, confirming the reinforcement of the passivation capability. Furthermore, the EEC model shown in Fig. 5b was used to interpret the EIS results, in which CPE_f_ and *R*_f_ represent the capacitance and resistance of the passive film, respectively. Fig. 9 shows the pH influences on the values of *R*_f_. From Fig. 9, the *R*_f_ value in each solution increased with increasing pH value, further confirming the reinforcement of the passivation capability. Fig. 10 shows the current transients of the Q235 samples in the HNO_3_–NaNO_2_, HAc–NaNO_2_ and HCl–NaNO_2_ solutions at the applied *E*_init_ potential. From Fig. 10, the area of the current transient in each solution decreases with increasing pH value, indicating a decrease in the passivation power (*Q*_pass_).^39^ In addition, the *i*_crit_ and *R*_f_ values at the same pH value in the HNO_3_–NaNO_2_, HAc–NaNO_2_ and HCl–NaNO_2_ solutions increased and decreased in turn, respectively (Fig. 7 and 9).

However, it is worth noting that the *i*_main_ value shown in Fig. 1 and the background current shown in Fig. 10 were independent of the pH value, suggesting the pH value only affected the passivation capability, but did not affect the actual passivation effectiveness.

## 4. Conclusions

For the Q235 carbon steel in the HNO_3_–NaNO_2_, HAc–NaNO_2_ and HCl–NaNO_2_ solutions, the influences of pH values on the manifestations of electrochemical characteristics and the variations of electrochemical parameters were discussed, and the following conclusions were obtained:

(1) The Q235 steel showed the A–P–T characteristic in the HNO_3_–NaNO_2_ solutions at pH 1 to pH 4 and in the HAc–NaNO_2_ solutions at pH 3 to pH 6, the sP-T characteristic in the HNO_3_–NaNO_2_ solutions at pH 5 and pH 6, the A characteristic in the HCl–NaNO_2_ solutions at pH 1 and pH 2, and the A–P–P characteristic in the HCl–NaNO_2_ solutions at pH 3 to pH 6.

(2) Except in the HCl–NaNO_2_ solutions at pH 1 and pH 2, the corrosion occurred on the Q235 surface with the positive shift of applied potential, followed by the occurrence of passivation. The influences of pH values on the corrosion and passivation behaviors were closely associated with the cathodic reactions of H^+^ reduction, O_2_ reduction and NO_2_^−^ reduction.

(3) In each solution, the corrosion rate decreased and the passivation capability strengthened with increasing pH value. At the same pH value, the corrosion rate increased and the passivation capability weakened in the HNO_3_–NaNO_2_, HAc–NaNO_2_ and HCl–NaNO_2_ solutions in turn, respectively.

## Conflicts of interest

There are no conflicts to declare.

## Supplementary Material
